# Evaluation of national fitness and national health development and coupling and coordination in 11 provinces and cities in Eastern China

**DOI:** 10.1371/journal.pone.0291515

**Published:** 2024-03-01

**Authors:** Dongxu Lin, Haixia Wang, Jinfu Xu, Lin Niu

**Affiliations:** 1 Sports Industry Development Research Centre, Fujian Jiangxia University, Fuzhou, China; 2 Handan Univeraity, College of Physical Education, Handan, China; 3 Fengfeng Mining District Committee Organization Department, Handan, China; Shanghai Dianji University, CHINA

## Abstract

Under the influence of development strategies with regard to national fitness and health in China, the interactive development between national fitness and national health is becoming increasingly strong. To explore the coupling and coordination relationship between national fitness and national health, this paper conducts an empirical analysis of the coupling and coordination relationship between national fitness and national health in 11 provinces and cities in Eastern China using the entropy weight method, a coupling coordination model, spatial visualization of the coupling coordination degree and spatial autocorrelation analysis. The research confirms that the comprehensive development level of national fitness and national health in Eastern China shows a steady upward trend, with a lag in national fitness as a whole, and that the growth rate of national fitness development is faster than that of national health development. The coupling coordination degree of the two systems of national fitness and national health in Eastern China shows a slow upward trend, and the coupling coordination degree rises from barely coordinated to primary coordination. There are significant differences in the spatial pattern of coupling coordination: the spatial pattern from north to south generally shows ‘low-high-high-low-high-low’ characteristics, and the spatial spillover effect of coupling coordination in various regions has not yet appeared. The revised GM(1.1) prediction results indicate that the level and improvement rate of coupling coordination will accelerate significantly in the next 10 years, but the spatial differences will still exist. Finally, suggestions are proposed to optimize the coupling and coordinated development of national fitness and national health based on policy guarantees as well as strengthening and cross-regional cooperation.

## Introduction

Since the 18th National Congress of the Communist Party of China (CPC), the Central Committee led by General Secretary, Xi Jinping has attached great importance to and exhibited concern for sports. It has exerted every effort to promote the reform and development of sports, and it has always prioritized people’s health. In 2014, the State Council’s opinions on accelerating the development of the sports industry and promoting sports consumption raised national fitness to the level of a national strategy [1]. In August 2016, General Secretary Xi Jinping proposed the following at the National Wellness Conference: ‘It is necessary to promote a healthy and civilized lifestyle, establish the concept of great hygiene and great health, transform disease treatment into people’s health, establish and improve the health education system, improve the health literacy of all people, and promote the deep integration of national fitness and national health’ [2]. In September 2020, in a speech to experts in the fields of education, culture, health and sports, Xi Jinping proposed ‘pushing forward the health barrier and establishing a new model of sports promotion with the cooperation of sports, health and other departments and the participation of the whole society’ [3]. This proposal further defines the direction of development and path of the two major strategies of national fitness and health.

## Literature review

### Definition of national fitness

To define the coupling of national fitness and national health, we first clarify their definitions. At present, national fitness and national health mainly have literal definitions. The formal proposal of national fitness can be traced back to the outline of the National Fitness Program promulgated by the Central People’s Government in 1995, in which national fitness refers to the people of the whole country, regardless of gender and age, with all people increasing their strength, flexibility, and endurance, improving their coordination and controlling the ability of all parts of the body, thus making people physically strong [4]. With the progress of the times, history is also providing us with new content for national fitness, and its definition is being extended by a large number of scholars. Dong held that national fitness is not merely words for China. Rather, it has developed into a cause of socialist construction, the sports practice of hundreds of millions of people, and a hot spot and unique social phenomenon related to sports at the end of the 20th century. The meaning of national fitness is not only literal, where people practice fitness, but also synonymous with the phrases "national fitness plan," "national fitness strategy," and "national fitness work" [5]. Qiu et al. [6] agreed that the development of national fitness can be promoted from the goal of ‘strengthening one’s physique’ to the new development stage of ‘health promotion’. Lu et al. [7] argued that national fitness is a large-scale social livelihood project led by the sports administrative department and that it covers all citizens. The implementation of relevant national policies and regulations on national fitness is taken as the main content of work to guide the public to participate in sports scientifically and to form a positive and healthy lifestyle by building a public fitness service system that meets the needs of the masses and ultimately achieves the goal of improving health levels.

### Definition of national health

The definition of universal health has no authoritative explanation in academic circles. With regard to health, since antiquity, China has believed that ‘no disease is healthy’. Cihai interprets health as a state in which the human body has normal functions, a well-developed organ system, energy, physical strength and good labor efficiency. Health is usually measured by anthropometry, physical examination and various physiological indicators. The World Health Organization (WHO) has proposed that ‘health is not only disease-free, but also includes physical health, mental health, good social adaptation and moral health.’ Lu et al. [7] reported that national health is a large-scale social livelihood project led by the Ministry of Health and that it covers all citizens. This project is the basic national policy of the country, with the implementation of relevant national health policies and regulations as the main content and with cobuilding and sharing as the basic path. The policy uses the reasonable control of various factors affecting health throughout the whole life cycle to achieve physical, mental and moral health, good social adaptation and, ultimately, national happiness.

### On the relationship between national fitness and national health

National fitness and national health have a strong correlation, and scholars are now beginning to pay attention to it and exploring it. Zhou et al. [8] held that national fitness is an action, while national health is the expected result of action, and there is a direct causal relationship between them. You [9] pointed out that the goal of national fitness is the realization of a healthy China, and national health takes the extensive development of national fitness as a means. Feng [10] believed that the deep integration and development of national fitness and national health need innovation in the system and mechanism; only when the two break down obstacles can we solve the current problems, such as insufficient infrastructure, unmatched facilities, the lack of professionals at the intersection of sports and medicine and low levels of management. Cui et al. [11] discussed the concept, characteristics and elements of national fitness and national health fusion symbiosis, affirming that the basis of national fitness and national health fusion symbiosis is the natural environment, the guarantee of fusion symbiosis is the artificial environment, and the key to fusion symbiosis is the social environment.

As an increasing number of countries are committing themselves to achieving national health coverage, the integration of fitness and health has evolved into an essential way for most countries to promote overall health [12]. Foreign research on national fitness and national health mainly includes three aspects: their importance, the relevant legal system for their integration, and the path of integration. In terms of the importance of national fitness and national health, Kujala et al. [13] showed that physical activity can help to improve physical fitness; reduce body fat, visceral fat and liver fat; increase the lumen diameters of conduit arteries to the lower limbs; increase bone mineral density in loaded bone areas; and increase the number of large high‐density lipoprotein particles. Min et al. [14] showed that skeletal muscle mass and physical activity are major modifiable factors that can moderate and prevent the occurrence of metabolic syndrome. Mazereel et al. [15] argued that physical activity has positive effects on self-esteem and sense of belongingness. The interactions between physical activity, self-esteem and belongingness are complex and fluctuate in everyday life. The results of Szychowska et al. [16] indicate that regular physical activity increases the chances of successful aging in older people, as physical activity lowers the risk of many chronic diseases and cognitive decline commonly associated with older age, promotes social engagement and improves self-estimated well-being. The US Department of Health and Human Services (HHS) regards physical activity as an important measure to prevent diseases [17]. In terms of the relevant legal system for the integration of national fitness and national health, Sharp et al. [18] proposed that population-level changes in physical activity may benefit from policy intervention. In response to the United Nations Sustainable Development Goals, Wales introduced legislation to holistically improve health and well-being, including public service boards, to improve the translation of national policy into practice [18]. Since the 1960s, the federal government of the United States has implemented the ‘Great Society’ program, which specifically involves supporting people participating in sports activities. The government has enacted a large number of sports bills, such as the Federal Water Recreation Act, Wild Scenic Area Act, Education Law Amendment Act, and National Adventure Trail Act [19]. In 2009, the Federal Personal Health Investment Proposal (PHIT) exempted citizens’ spending on mass sports and fitness from consumption tax, encouraging more people to participate in sports [20]. In terms of the integration of national fitness and national health, Krejza (2007) summarized the effectiveness of the European Union (EU) White Paper on Sports, noting that the EU has integrated sports elements into 53 policy projects and compiled them as the Pierre de Coubertin Action Plan, which provides considerable information on the rule-of-law system for the integration of public fitness and health [21]. The National Physical Activity Program developed by the National Physical Activity Alliance (NPAP) has formulated action strategies for six departments in addition to mass media and nonprofit organizations to assist in implementation [22]. To promote physical activity among children and adolescents, the Presidential Fitness, Exercise and Nutrition Commission (PCFSN) works with various organizations to carry out related programs, including the Presidential Challenge Program, the Presidential Active Life Award and the Presidential Youth Fitness Program, dedicated to educating and motivating Americans to live in a healthy way [23].

In summary, national fitness and national health have always been hot topics. Most previous studies have analyzed the positive role of national fitness and national health in promoting national construction, in realizing a happy life for people, in promoting the healthy growth of children and adolescents and in improving national physical quality and health. The mutual promoting relationship between the two is also widely recognized. Existing research, unfortunately, still has the following shortcomings: in terms of research methods, most studies are qualitative research, and there is almost no empirical research on the interaction between national fitness and national health systems based on data mining and model construction. In terms of research content, studies mainly focus on the systematic relationship between national fitness, the national health and social environment, the regional economy, the sports industry, the public service system, healthy China construction, etc. However, the discussion on the coupling relationship between national fitness and national health has been ignored. It is worth noting that the interaction between the two has a direct impact on the construction of a healthy China. In this study area, research is mostly limited to a single city or the level of the nation as a whole, and there is almost no comparison between different provinces and cities in different regions. However, the strategic focus of China’s Belt and Road Initiative and the Yangtze River Economic Belt and the coordinated development of Beijing, Tianjin and Hebei are precisely the complementary advantages and coordinated development in the region, providing an expandable space for this study and a theoretical reference for clarifying the underlying logic.

Accordingly, to comprehensively explore the current situation of the coupling development of national fitness and national health, a coupling coordination evaluation index system for national fitness and national health is constructed on the basis of existing theoretical research and is combined with panel data on national fitness and national health indicators covering 11 provinces and municipalities directly under the central government in Eastern China. Subsequently, the entropy weight method and coupling coordination degree model are used to explore the comprehensive development level and coupling coordination degree of national fitness and national health in 11 provinces and cities in Eastern China from 2016 to 2020. The coupling and coordinated development of the two systems in the eastern region in the next decade is predicted. Eastern China is part of a key strategic plan made by the CPC based on the reality of China’s development and the inherent laws of China’s economy. Since the 1980s, under the guidance of the policy of ‘reform and opening up, the east first’, the eastern region has developed into China’s most active coastal urban belt, attracting the most foreign investment, the largest economic output and the largest international influence. All these factors indirectly imply that studying the coupling of national fitness and national health in Eastern China will have a reasonable and effective demonstration effect on the development of other regions.

Compared with existing studies, the possible marginal contributions of this study are as follows: first, in the study area, with respect to the comprehensive development level of national fitness and national health in different provinces and cities in Eastern China, the present situation and shortcomings of their coupling and coordination are further identified, which is beneficial for more accurately promoting the coordinated development of national fitness and national health. Second, the first coupling coordination evaluation index system for national fitness and national health is constructed. This system provides a better underlying logic and basis for follow-up related research. Third, the coupling coordinated development of national fitness and national health is explored based on the coupling coordination degree model, which enriches frontier research on national fitness and national health. In addition, it provides a two-way decision-making basis for the current concept in which ‘national fitness drives the development of national health and national health feeds back the development of national fitness’, and it can serve as a basis for follow-up basis and experience support.

## Methodology

### Entropy weight method

The methods of determining weights are mainly divided into two categories, namely, subjective and objective weighting methods. Among them, the analytic hierarchy process method of subjective weighting is widely used. However, the AHP has apparent subjective tendencies, and the final result based on the determined weight has no practical reference value [24]. To ensure the objectivity, correctness and scientificity of the evaluation results, the entropy weight method is adopted. The reason is that this method is not affected by the linear correlation of the evaluation index. It determines the weight based on index variability and the meaning of the value itself. Thus, the interference of human factors can be avoided, and the weighting process is transparent and reproducible. Therefore, the weight has high credibility [25, 26]. The specific steps of the entropy weight method are as follows:

Step 1: The dimensions and units of each index differ because they cannot be directly compared and calculated. Therefore, these items need standardization before the index weight is calculated [27]. When the index is positive, the standardized calculation formula is:

$$x_{ij}^{\prime }=\frac{x_{ij}-x_{j}^{min}}{x_{j}^{max}-x_{j}^{min}}$$

When the index is negative, the standardized calculation formula is:

$$x_{ij}^{\prime }=\frac{x_{j}^{max}-x_{ij}}{x_{j}^{max}-x_{j}^{min}}$$

where $x_{ij}^{\prime }$ represents the standardized data of the indicator of the first year, $x_{j}^{max}$ represents the maximum value of the indicator, x_ij_ represents the actual value of the indicator of the first year, and $x_{j}^{min}$ represents the minimum value of the indicator.

Step 2: After standardization, the value of the individual index has no calculation significance because the minimum value of the standardized data is 0 when the weight is calculated by the entropy weight method. To eliminate this situation, we must translate the standardized value. The translation formula is $x_{ij}^{\prime \prime }=H+x_{ij}^{\prime }$, wherein H represents the magnitude of the numerical translation, usually taking the value of 1.

Step 3: The data after translation are made dimensionless by the specific gravity method, and the specific gravity of each evaluation index is calculated:

$$y_{ij}=\frac{x_{ij}^{\prime \prime }}{\sum_{i=1}^{n}x_{ij}^{\prime \prime }}$$

Step 4: The entropy value of the j-th index is calculated by the entropy calculation formula: $e_{j}=-\frac{1}{\ln\;n}\sum_{i=1}^{n}y_{ij}\;\ln\;y_{ij}$.

Step 5: The difference coefficient of the j-th index is calculated by the difference coefficient calculation formula:

$$g_{j}=1-e_{j}.$$

Step 6: The weight value of the j-th index is calculated by the weight calculation formula, wherein j = 1,2,⋯ ⋯n, and n is the number of indicators.


$$\omega_{j}=\frac{g_{j}}{\sum_{j=1}^{n}g_{j}}$$

Step 7: The index of the comprehensive development level of national fitness and national health is calculated through the weight value of each index and standardized data. The calculation formula is: $Z_{i}=\sum_{j=1}^{p}\omega_{j}x_{ij}^{\prime }$

### Coupling coordination model

The United Nations proposed Sustainable Development Goals, requiring us to enhance resource-use efficiency and promote sustainable lifestyles [28]. Hence, measuring the coordinated interactions between national fitness and national health with the concept of sustainability is an important and useful approach [29]. To analyze the coupling effect and the degree of coordination between the integration of national fitness and national health and sustainable development, we use the concept and principle of coupling in physics. The two systems of national fitness and national health have a strong correlation and permeability, and thus, their relationship can be studied by using coupling coordination theory. This model can reflect the system function and structure, and thus, it can be used to describe the influence and interaction of different elements and systems. Both systems can form a coupling coordination mechanism wherein both support and hinder one another and gradually evolve to a benign interactive status [30]. The coupling coordination degree model has apparent advantages in analyzing complex systems, including coupling and coordination degrees [31]; the former is used to measure the strong interaction between systems, and the latter reflects the relationship of coordination and the virtuous circle between systems [32].

Calculate the coupling degree, where U and G represent the index of the comprehensive development level of national fitness and national health, respectively.


$$C=2{\left[\frac{U\times G}{{\left(U+G\right)}^{2}}\right]}^{\frac{1}{2}}$$

Calculate the coupling coordination degree, where D represents the coupling coordination degree of the two systems, and the value range is 0–1. T represents the comprehensive evaluation index of national fitness and national health, and T = αU+βG, wherein α and β represent the weight of national fitness and national health, respectively, and α+β = 1. Based on the advice of the experts consulted, α = 0.5 and β = 0.5.


$$D=\sqrt{C\times T}$$

To directly reflect the coupling relationship between national fitness and national health, we classify the coupling coordination degree of the two systems by referring to the division method of coupling coordination degrees of Yang et al. [33], as shown in Table 1.

**Table 1:** Grading standard of the coupling coordination degree between national fitness and national health.

No.	Coupling coordination degree (D value)	Coordination degree grade	No.	Coupling coordination degree (D value)	Coordination degree grade
1	0.00–0.1	Extreme maladjustment	6	0.5001–0.6	Barely coordinated
2	0.1001–0.2	Severe maladjustment	7	0.6001–0.7	Primary coordination
3	0.2001–0.3	Moderate maladjustment	8	0.7001–0.8	Intermediate coordination
4	0.3001–0.4	Mild maladjustment	9	0.8001–0.9	Good coordination
5	0.4001–0.5	Imminent maladjustment	10	0.9001–1.00	High-quality coordination

## Construction of the index system

The data in this study are derived from the China Health Statistical Yearbook, the China Environmental Statistical Yearbook, the China Tertiary Industry Statistical Yearbook, the China Statistical Yearbook and the official website of the Ministry of Finance of China.

The link between national fitness and national health is not a one-to-one corresponding linear relationship; that is, their interaction is more complex. Research on the coupling coordination of complex systems with coupling interactions must be carried out on the basis of a multi-index comprehensive evaluation of the two systems [34]. To explore the degree of coordination between national fitness and national health, an index system is constructed on the basis of analyzing the connotation of their correlation model and following the principles of systematicity, typicality, relevance and availability. The relevant indicators are screened and determined through three steps: frequency analysis, theoretical analysis and expert consultation. First, frequency analysis is used. Specifically, index systems for national fitness and national health are searched in the China National Knowledge Infrastructure (CNKI) database, the indicators used in the literature are counted, and the indicators with the highest frequency are selected to form the first round of the index set. Second, theoretical analysis is adopted with reference to the division methods of the national fitness evaluation indicators of Zhan et al. [35], Shi et al. [36] and Pu et al. [37]. Recently, it has not been possible to fully obtain Chinese data on national fitness-related indicators. Since the cause of national fitness is an integral part of the tertiary industry and an important means of protecting and developing productive forces and because data on the culture, sports and entertainment industry are complete, the indicators of the culture, sports and entertainment industry are used as part of the evaluation of national fitness, a method that is widely used in the literature [38]. At the same time, the evaluation index system of healthy China construction in the Outline of Healthy China 2030 and the National Healthy City Evaluation Index System (2018 Edition) is selected as part of the national health evaluation index. Finally, the constructed indicators are sent to 10 field experts as part of the consultation method, and the indicators are further optimized and adjusted. Based on the steps above, 18 individual indicators are subdivided into six dimensions: the national fitness environment, national fitness participation, national fitness investment, health environment construction, health education promotion and healthy population development. Moreover, a coupling coordination evaluation system for national fitness and national health is established (Table 2).

**Table 2:** Evaluation index system for the coupling coordination between national fitness and national health.

System layer	Dimension layer	Index layer	Weight	Data source
National fitness	National fitness environment	City park (unit)	0111	Statistical Yearbook of China’s Tertiary Industry
		Sports coaches (people)	0113	Statistical Yearbook of China’s Tertiary Industry
		Number of legal entities in the culture/sports/entertainment industry (units)	0094	Statistical Yearbook of China’s Tertiary Industry
	Participation in national fitness	Retail income of cultural/sporting goods and equipment (100 million yuan)	0126	China Economic Census Yearbook
		Business income of corporate units in the culture/sports/entertainment industry (100 million yuan)	0096	Statistical Yearbook of China’s Tertiary Industry
		Employment in urban units of the culture/sports/entertainment industry (10,000)	0120	Statistical Yearbook of China’s Tertiary Industry
	National fitness investment	Sports lottery public welfare fund expenditure (10,000 yuan)	0109	Official website of the Ministry of Finance of China
		Investment in fixed assets of the whole society in the culture/sports/entertainment industry (100 million yuan)	0143	Statistical Yearbook of China’s Tertiary Industry
		Local finance culture/sports/media expenditure (100 million yuan)	0084	China Statistical Yearbook
National Health	Healthy environment construction	Urban green coverage area (ha)	0099	China Environmental Statistics Yearbook
		Total investment in urban environmental infrastructure construction (100 million yuan)	0113	China Environmental Statistics Yearbook
		Number of beds in primary medical and health institutions (Zhang)	0150	China Health Statistics Yearbook
	Health education promotion	Public health education activities (II)	0108	China Health Statistics Yearbook
		Number of health education trainers (person-times)	0088	China Health Statistics Yearbook
		Number of special education schools (institutes)	0139	China Statistical Yearbook
	Healthy population development	Average life expectancy (years)	0127	China Health Statistics Yearbook
		Incidence of class A and B infectious diseases per 100,000 population (per person)	0069	China Health Statistics Yearbook
		Number of people admitted to primary medical and health institutions (10,000)	0102	China Health Statistics Yearbook

## Results and analysis

### Analysis of the comprehensive development level of national fitness and national health in Eastern China

**Fig 1:**
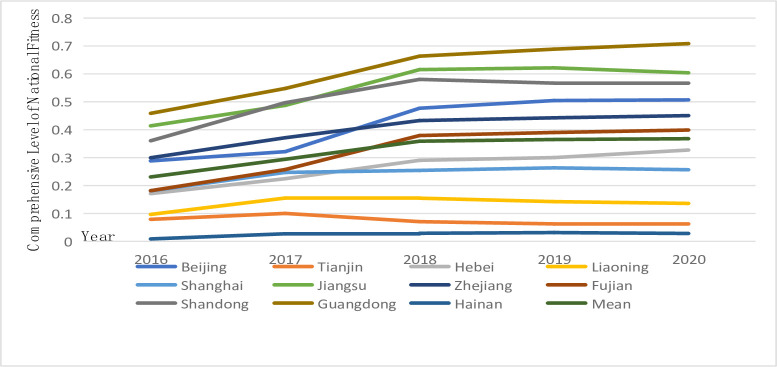
Development trend of the comprehensive level of national fitness in 11 provinces and cities in Eastern China.

**Fig 2:**
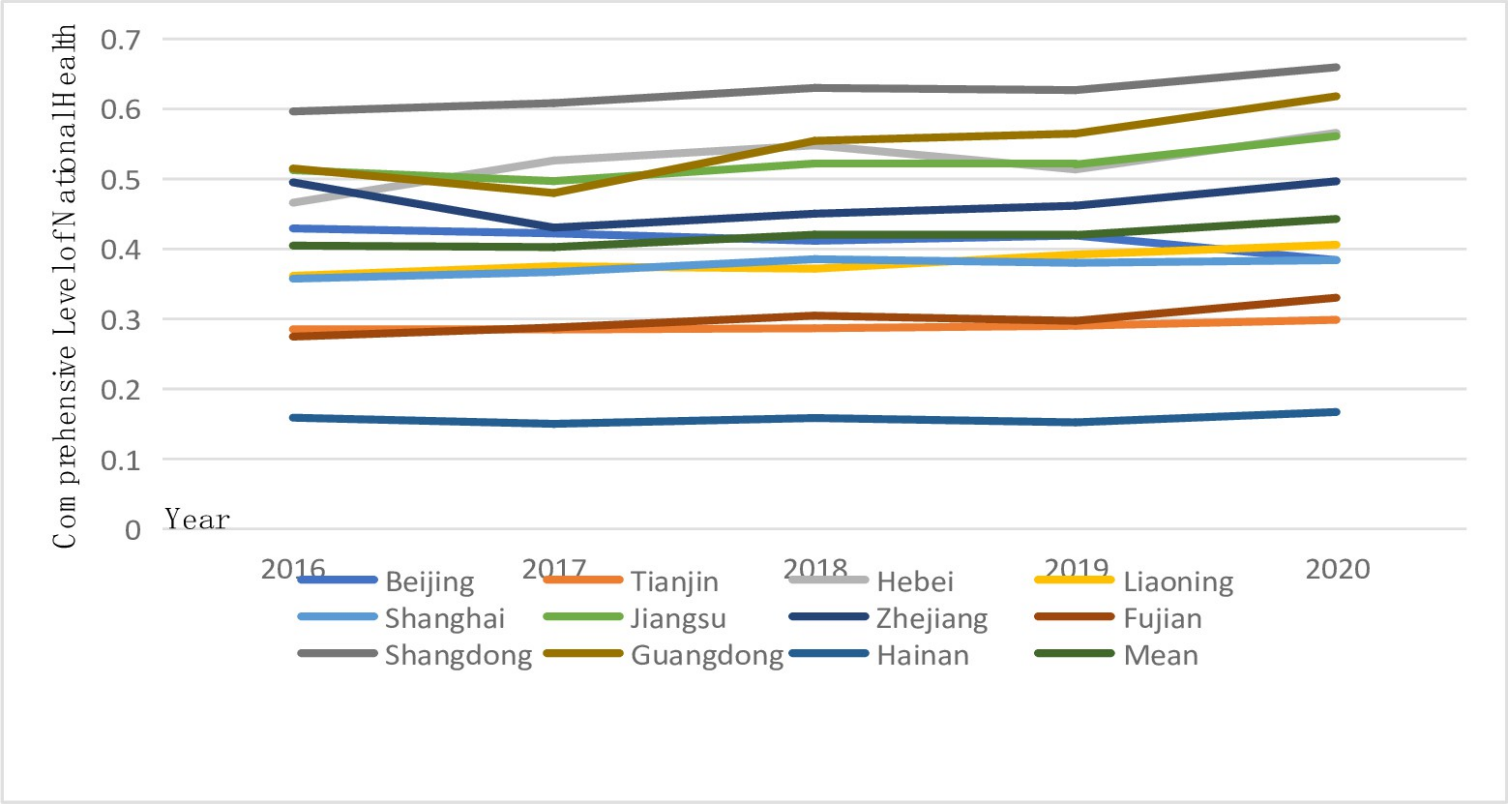
Development trend of the comprehensive level of national health in 11 provinces and cities in Eastern China.

According to Figs 1 and 2, the development of the national fitness and national health systems in Eastern China exhibits considerable similarities. On the whole, the development of national fitness and the comprehensive level of national health show an upward trend, but for several provinces and cities in 2017, the trend fluctuated to varying degrees. These fluctuations may have been caused by the external environment of slowing world economic growth in 2017 and the internal factors of the reduction in sports-related fixed asset investment in several parts of China. As a result, the comprehensive development level of national fitness and national health in several provinces and cities fluctuated downward. The development of the comprehensive level of national fitness and national health shows that Guangdong, Shandong and Jiangsu are in the lead, while Tianjin and Hainan are at the bottom of the stratification, which also reflects the high correlation between the two systems. Internal functions also promote and complement each other; however, the two systems of national fitness and national health in Eastern China also show significant spatial differences. In Guangdong, Shandong, and Jiangsu provinces, factors such as socioeconomic strength, infrastructure conditions, the level of science and technology education, human resources, and the degree of openness to the outside world have a significant impact on the rapid development of the comprehensive level of national fitness and health. Guangdong, Shandong, and Jiangsu are three of China’s most economically developed provinces. As the pioneering provinces of China’s reform and opening up, they have successfully established a distinctive pattern of promoting the development of national fitness and health through reform and opening up. It is important to note that Tianjin’s overall level of national fitness has shown negative growth. As a result, in the ensuing development process, on the basis of coordinating the development of national fitness, Tianjin’s tilting of supporting finances, policies, and health service talent should be strengthened to ensure the balanced development of the internal structure of national fitness.

Figs 1 and 2 suggest variations in the dynamic characteristics of the comprehensive level of national fitness and national health. From the curve of the development level of the two major systems, the fluctuation in national fitness in Eastern China is strong, the fluctuation range of the comprehensive level of national health is relatively small, and the development is relatively moderate. These results indicate that it is difficult to significantly change the national health level of 11 provinces and cities in Eastern China in a short period of time, while the national fitness system with its wide range, strong foundation and high correlation has become an effective means of narrowing regional differences. According to the average development level of national fitness and national health, the comprehensive level of national fitness in Eastern China from 2016 to 2020 was lower than that of national health, with a lag in national fitness development. In other words, national fitness has a relatively weak driving effect on the development of national health, which in turn plays an obvious role in promoting the development of national fitness. The average comprehensive development level of the two systems increased from 0.230840 and 0.404773 in 2016 to 0.367804 and 0.442872 in 2020, respectively. Clearly, the development level of national fitness in Eastern China has gradually approached the level of national health development, indicating that the development of national fitness is gradually playing a role in driving national health. This result demonstrates the importance that the Central Committee of the CPC, led by Comrade Xi Jinping, places on building a healthy China, emphasizing people’s health in the context of development strategy, and obtaining outstanding results in the work of ensuring everyone’s fitness and health.

U and G represent the index of the comprehensive level of national fitness and national health development, respectively, when examining whether the comprehensive level of national fitness and national health development is synchronized. If U > G, then the development of national fitness is better than that of national health, indicating a lag in national health. In contrast, if U < G, the development of national fitness is worse than that of national health, indicating a lag in national fitness development.

According to Table 3, the number of provinces and cities in Eastern China from 2016 to 2020 with a lag in national fitness is more than that with a lag in national health, which re-emphasizes that the comprehensive level of national health in Eastern China is better than that of national fitness. According to comparative analysis, seven provinces and cities, i.e., Tianjin, Hebei, Liaoning, Shanghai, Zhejiang, Shandong and Hainan, all show a lag in national fitness; that is, the driving effect of national fitness on national health is not obvious. Correspondingly, Beijing, Jiangsu, Fujian and Guangdong show a lag in national fitness and national health, and they all develop from a lag in national fitness to a lag in national health. These results indicate the improving national fitness work in these four provinces and cities, thereby driving the development of the national health system. In the specific implementation category of the guiding ideology, the attention of inland provinces is more focused on the fulfillment of the masses’ demand for sports and fitness in China, whereas coastal provinces, such as Guangdong, Jiangsu, and Fujian, have already met the masses’ demand for sports and fitness to a certain degree and are more focused on the in-depth integration of fitness for all.

**Table 3:** Relative development types of national fitness and national health.

Year	2016	2017	2018	2019	2020
Beijing	Lag in Fitness	Lag in Fitness	Lag in Health	Lag in Health	Lag in Health
Tianjin	Lag in Fitness	Lag in Fitness	Lag in Fitness	Lag in Fitness	Lag in Fitness
Hebei	Lag in Fitness	Lag in Fitness	Lag in Fitness	Lag in Fitness	Lag in Fitness
Liaoning	Lag in Fitness	Lag in Fitness	Lag in Fitness	Lag in Fitness	Lag in Fitness
Shanghai	Lag in Fitness	Lag in Fitness	Lag in Fitness	Lag in Fitness	Lag in Fitness
Jiangsu	Lag in Fitness	Lag in Fitness	Lag in Health	Lag in Health	Lag in Health
Zhejiang	Lag in Fitness	Lag in Fitness	Lag in Fitness	Lag in Fitness	Lag in Fitness
Fujian	Lag in Fitness	Lag in Fitness	Lag in Health	Lag in Health	Lag in Health
Shandong	Lag in Fitness	Lag in Fitness	Lag in Fitness	Lag in Fitness	Lag in Fitness
Guangdong	Lag in Fitness	Lag in Health	Lag in Health	Lag in Health	Lag in Health
Hainan	Lag in Fitness	Lag in Fitness	Lag in Fitness	Lag in Fitness	Lag in Fitness

### Grade analysis of the coupling coordination degree between national fitness and national health in Eastern China

Table 4 shows that the coupling coordination degree of national fitness and national health in Eastern China increased from 0.526 in 2016 to 0.604 in 2020. Additionally, the level of coupling coordination changed from barely coordinated to primary coordination. This promotion directly confirms the continuous strengthening of the interaction between the two systems and that their coupling and coordination relationship is developing in a benign manner. From the average value of coupling coordination, Guangdong, Shandong and Jiangsu have a moderate coupling coordination degree, while Zhejiang, Beijing and Hebei are at the primary coordination level. The main reason for the variations is that Guangdong Province is not only the largest in the national economy but also a well-known province that is strong in sports in China. Guangdong Province has three professional Chinese men’s basketball league teams and four Chinese Football Association Super League teams. These facts indirectly imply that Guangdong sports play an important role in China’s sports territory and that the strategic position of the province determines its role in contributing Guangdong’s strength to the construction of national fitness and national health. Meanwhile, Shandong Province insists on sports to benefit people and includes national fitness in the government work report, where national fitness is included in the key work of Healthy Shandong to ensure that the province is strong in sports and rural revitalization. Mass sports in the province are characterized by the large-scale construction of facilities, the wide coverage of sports services and the high number of fitness activities. The main indicators of the service system are at the forefront of the country. Jiangsu Province takes the lead in proposing and building a ‘10-minute sports fitness circle’ in urban communities, achieving full coverage of sports facilities in administrative villages, and building the only demonstration area of the national public sports service system with the province as a unit. This series of measures lays the foundation for the interaction between national fitness and national health. Therefore, the three provinces rely on their own unique advantages to enable the interaction between and the promotion of the two systems, which has a positive effect on their coupling and coordination. The provinces with barely coordinated coupling coordination are Fujian and Shanghai. Liaoning is on the verge of maladjustment, Tianjin is mildly maladjusted, and Hainan is moderately maladjusted.

**Table 4:** Coupling coordination degree of national fitness and national health.

Year	2016	2017	2018	2019	2020	Mean
Guangdong	0.697158	0.71606	0.778844	0.789723	0.813486	0.759054
Shandong	0.680950	0.741451	0.777625	0.772111	0.781734	0.750774
Jiangsu	0.678663	0.701492	0.752882	0.75428	0.762971	0.730058
Zhejiang	0.620624	0.632284	0.66446	0.672396	0.687795	0.655512
Beijing	0.593170	0.606983	0.665681	0.678132	0.664253	0.641644
Hebei	0.531345	0.586297	0.631615	0.62663	0.655967	0.606371
Fujian	0.472609	0.521607	0.583037	0.583545	0.602466	0.552653
Shanghai	0.504582	0.548769	0.559495	0.562569	0.560267	0.547136
Liaoning	0.432293	0.491281	0.489767	0.486057	0.484789	0.476837
Tianjin	0.387338	0.411014	0.377316	0.367191	0.370110	0.382594
Hainan	0.193154	0.252816	0.260367	0.263197	0.262397	0.246386
Mean	0.526535	0.564551	0.594644	0.595985	0.604203	0.577184

**Fig 3:**
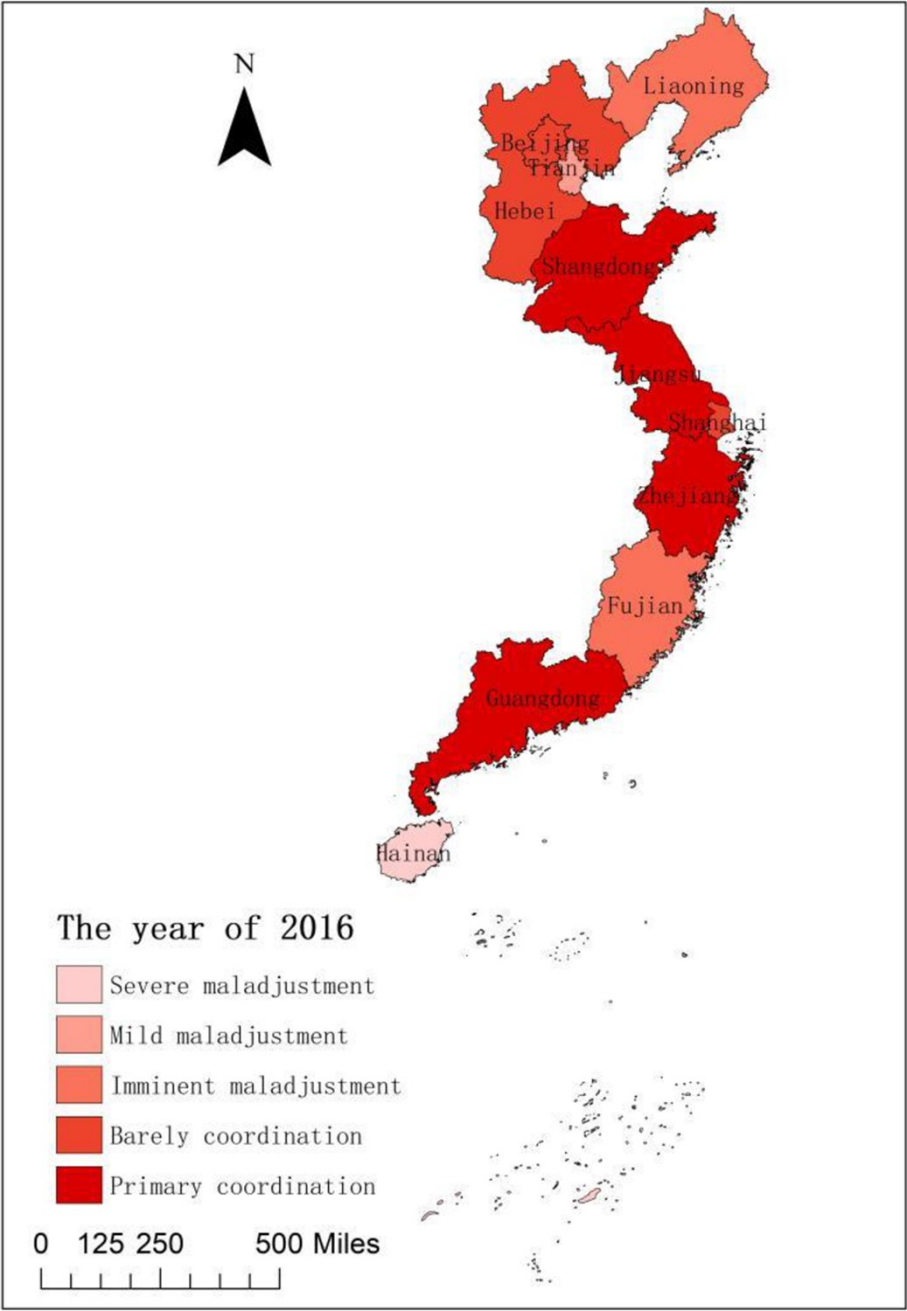
Spatial difference in the coupling coordination degrees between 11 provinces and cities in Eastern China in 2016.

**Fig 4:**
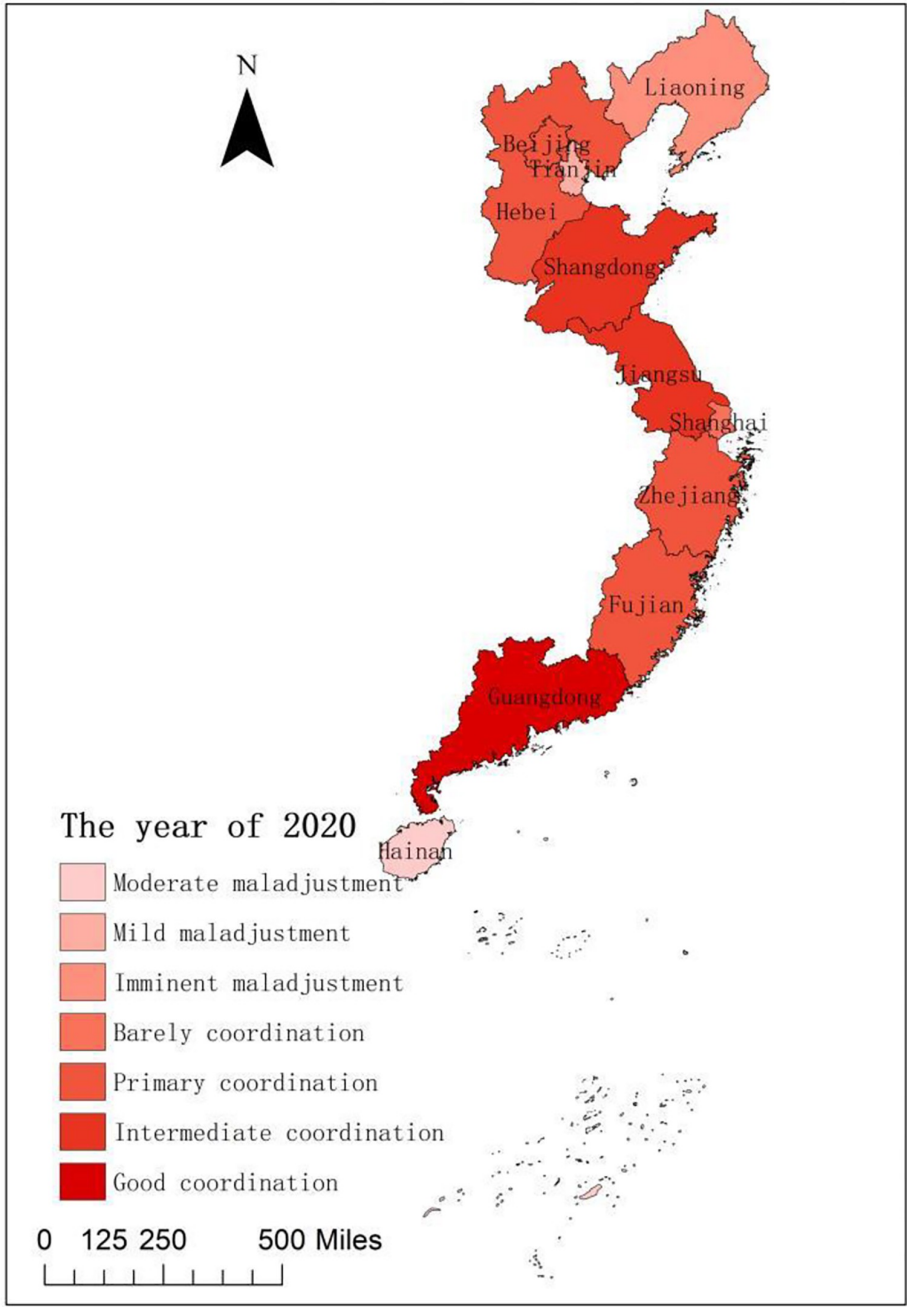
Spatial difference in the coupling coordination degrees between 11 provinces and cities in Eastern China in 2020.

To compare the coupling coordination of national fitness and national health development in 11 provinces and cities in Eastern China, we spatially visualize the coupling coordination degrees of national fitness and national health in 11 provinces and cities in Eastern China in 2016 and 2020 using ArcGIS 10.2 software. The temporal and spatial comparisons among regions give us new prospects to discover the coordinated growth relationships between the two systems [39]. Fig 3 shows a great spatial difference in the coupling coordination degree of national fitness and national health development among the cities and provinces in the sample in 2016. The spatial pattern from north to south generally shows a ‘low-slightly high-high-low-low-low’ trend. In other words, the trend transitioned from imminent maladjustment in Liaoning to barely coordinated between Beijing and Hebei to mild maladjustment in Tianjin. The trend also transitioned from primary coordination in Shandong, Jiangsu and Zhejiang to barely coordinated in Shanghai, to imminent maladjustment in Fujian, o the primary coordination in Guangdong and finally to serious maladjustment in Hainan. Fig 4 indicates that, except for Tianjin, Liaoning, Shanghai and Zhejiang, the coupling coordination degrees of national fitness and national health in 11 provinces and cities in Eastern China in 2020 are the same as those in 2016. The distribution pattern is basically the same, and the spatial difference remains. Guangdong has been upgraded from primary to good coordination, indicating that after five years of development, the integration of the two major systems of national fitness and national health in Guangdong Province has relatively matured. Fujian is on the verge of maladjustment and has been upgraded by two levels to primary coordination at a remarkable rate of growth. Beijing and Hebei have been promoted from reluctant to primary coordination, while Jiangsu and Shandong have been promoted from primary to intermediate coordination. Hainan has increased from severe to moderate maladjustment. In the past five years, Hainan has constructed a ‘National Fitness +’ multidimensional comprehensive innovation construction model of by giving full play to its own advantages, and it has accelerated deep ‘sports + tourism’ integration. The coordinated development of mass and competitive sports is promoted by holding high-level sports competitions, such as the Open Golf Race, the Island Ring Race and the Yacht Race. Fundamentally, the coupling and coordination of national fitness and national health are in the initial stage of development.

**Fig 5:**
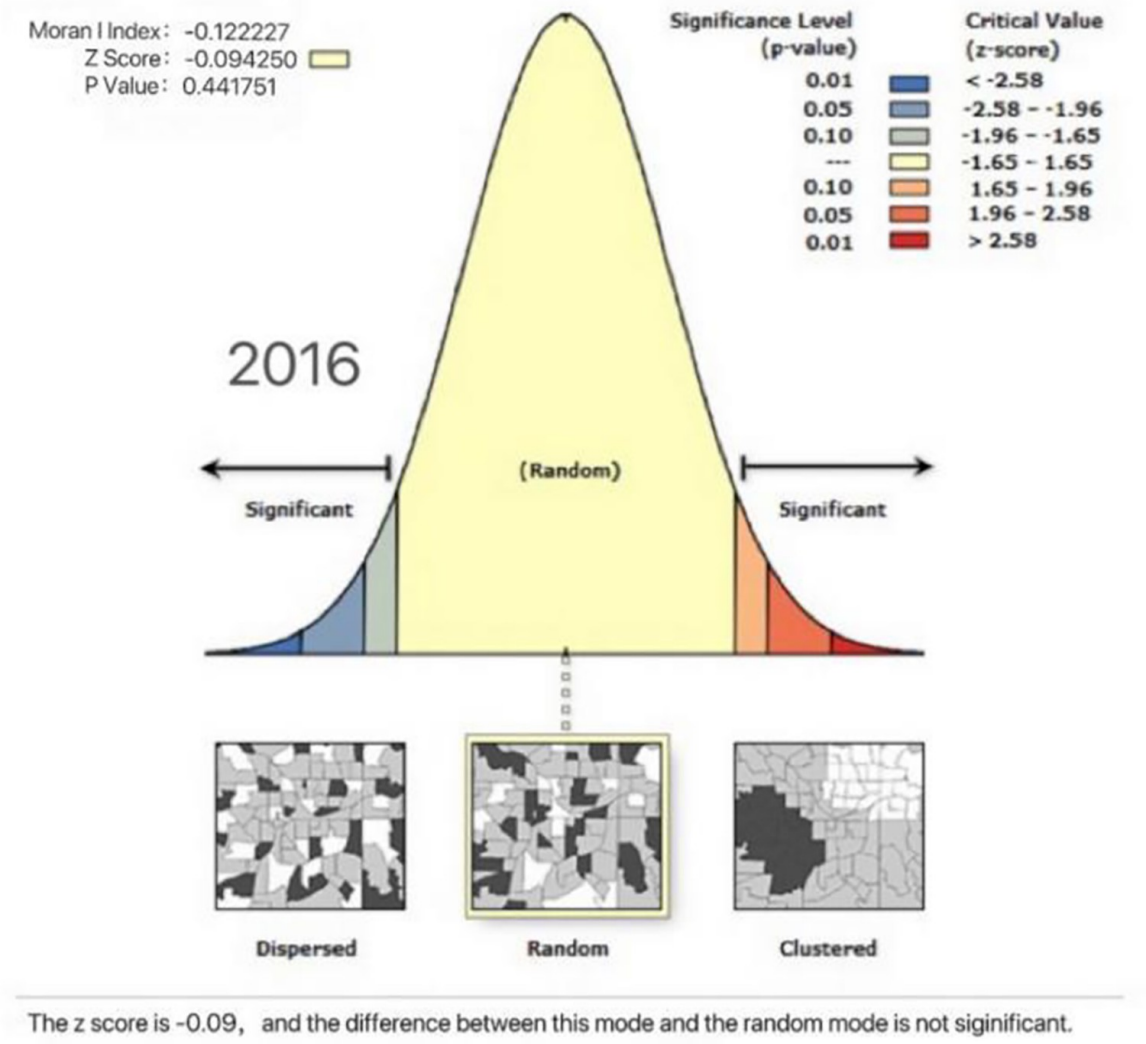
Spatial autocorrelation of the coupling coordination degree of the two major systems in 11 provinces and cities in Eastern China in 2016.

**Fig 6:**
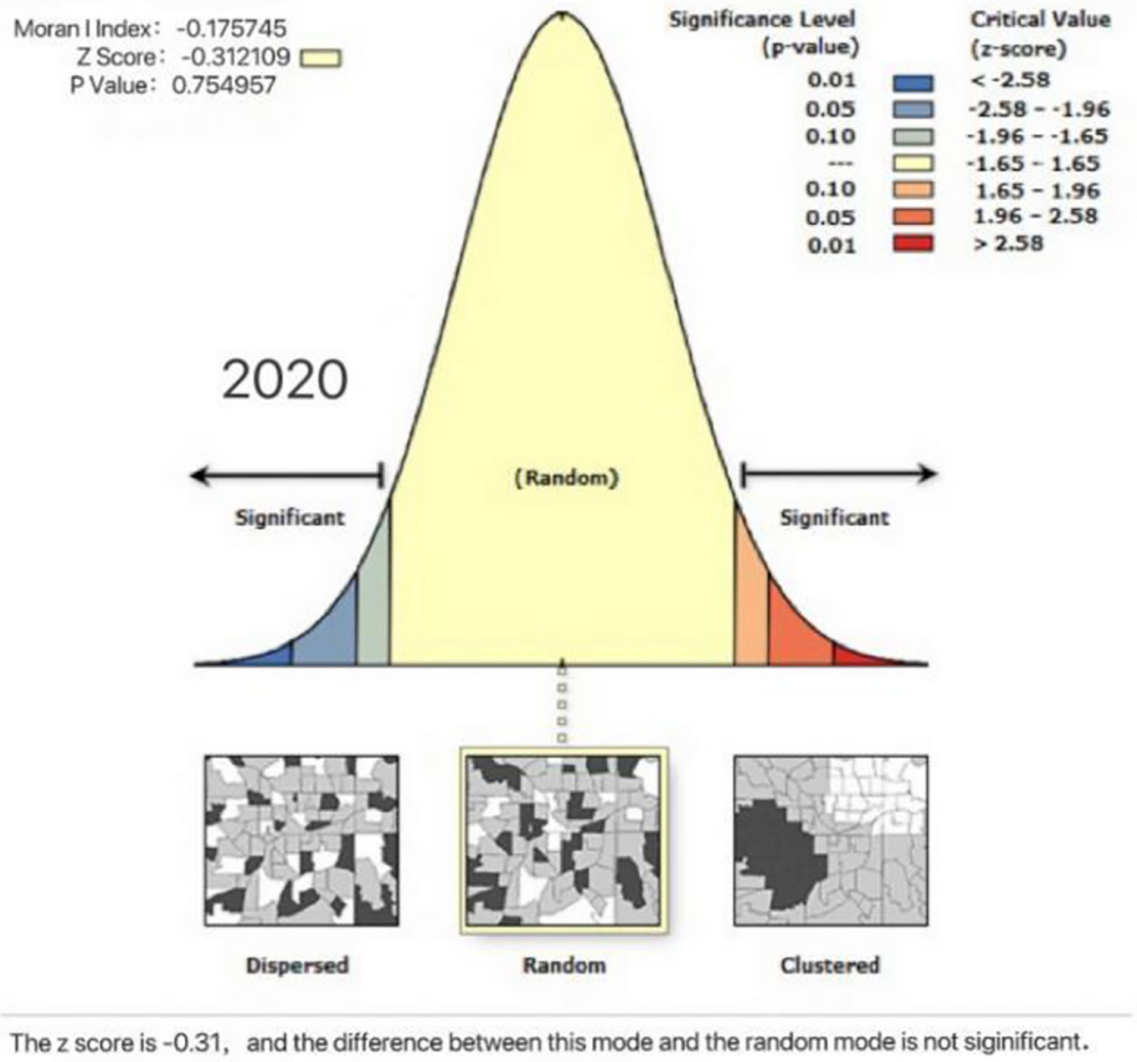
Spatial autocorrelation of the coupling coordination degree of the two major systems in 11 provinces and cities in Eastern China in 2020.

To explore the spatial correlation between the coupling coordination degree of national fitness and national health in Eastern China, we calculate the Moran index of the coupling coordination degree of the two systems in 2016 and 2020 using ArcGIS 10.2 software. If the value is greater than 0, then there is a positive spatial correlation; conversely, if the value is less than 0, there is a negative spatial correlation; and if the value is close to 0, the degree of spatial correlation is low or shows a random distribution [40]. From Figs 5 and 6 the Moran indices of 2016 and 2020 are -0.1222 and -0.1757, and the z scores are -0.0942 and -0.3121, respectively, indicating that the coupling coordination degree of national fitness and national health in Eastern China is random in space and has an insufficient spatial spillover effect and no spatial aggregation, a phenomenon resulting from their own development and that has existed for a long time.

### Forecast of the coordinated development degree of coupling between national fitness and national health in Eastern China

Taking the coupling coordination degree of national fitness and national health in Eastern China from 2016 to 2020 as the data analyzed, the development of their coupling coordination degree is predicted using DPS analysis software on the basis of the gray GM(1.1) prediction model. This model applies especially when the sample involves limited data [41]. The prediction parameters are set, the residual repeated modeling times are set to seven, the prediction time length is one, and the predicted value of the coupling coordinated degree of national fitness and national health in 2021 is calculated. Subsequently, a new data series is generated from the predicted value for 2021 and the original value from 2016 to 2020. Then, the predicted value of the coupling coordinated degree of national fitness and national health in 2022 is calculated based on the previous residual repeated modeling times and prediction time length, and the steps above are repeated. Finally, the prediction of the coupling coordination degree of national fitness and national health in 11 provinces and cities in Eastern China from 2020 to 2030 is obtained. Table 5 shows that the coupling coordination degree of national fitness and national health in Eastern China will change from barely coordinated to the intermediate coordination stage from 2020 to 2030, showing rapid evolution and promotion. However, spatial differences between regions are still inevitable. With the publication of a series of policies and regulations, including the People’s Republic of China’s Sports Law (revised in 2022), the Outline of the Fourteenth Five-Year Plan for the National Economic and Social Development of the People’s Republic of China, Vision 2035, and the Outline of the "Healthy China 2030" Plan, fitness for all and universal health have gained increasing attention and support in public policy. People’s fitness and health needs will be better met. As a result, it is predicted that throughout the 2020–2030 period, with the successful execution of various policies, the two systems will harmonize and promote each other and continue to develop in a better and more orderly path.

**Table 5:** Forecast of the coupling and coordinated development of the two major systems in 11 provinces and cities in Eastern China.

Year	Beijing	Tianjin	Hebei	Liaoning	Shanghai	Jiangsu	Zhejiang	Fujian	Shandong	Guangdong	Hainan	Mean
2016	0.593	0.387	0.531	0.432	0.505	0.679	0.621	0.473	0.681	0.697	0.193	0.527
2017	0.607	0.411	0.586	0.491	0.549	0.701	0.632	0.522	0.741	0.716	0.253	0.565
2018	0.666	0.377	0.632	0.490	0.559	0.753	0.664	0.583	0.778	0.779	0.260	0.595
2019	0.678	0.367	0.627	0.486	0.563	0.754	0.672	0.584	0.772	0.790	0.263	0.596
2020	0.664	0.370	0.656	0.485	0.560	0.763	0.688	0.602	0.782	0.813	0.262	0.604
2021	0.700	0.349	0.677	0.482	0.567	0.790	0.709	0.635	0.797	0.852	0.268	0.621
2022	0.720	0.337	0.700	0.480	0.571	0.810	0.727	0.662	0.809	0.886	0.271	0.634
2023	0.740	0.325	0.723	0.478	0.575	0.830	0.747	0.690	0.821	0.921	0.274	0.648
2024	0.760	0.313	0.747	0.475	0.579	0.851	0.767	0.720	0.834	0.957	0.278	0.662
2025	0.782	0.303	0.771	0.473	0.583	0.872	0.787	0.751	0.846	0.995	0.281	0.677
2026	0.803	0.292	0.797	0.471	0.587	0.894	0.808	0.783	0.859	1.035	0.284	0.692
2027	0.826	0.282	0.823	0.469	0.590	0.916	0.829	0.816	0.872	1.075	0.288	0.708
2028	0.849	0.272	0.850	0.466	0.594	0.939	0.851	0.851	0.885	1.118	0.291	0.724
2029	0.873	0.262	0.878	0.464	0.598	0.963	0.874	0.887	0.898	1.162	0.295	0.741
2030	0.897	0.253	0.907	0.462	0.602	0.987	0.897	0.925	0.912	1.208	0.298	0.759

From a provincial perspective, all provinces will improve greatly on the whole. The coupling coordination degrees of Guangdong, Jiangsu, Fujian, Shandong and Hebei will enter the stage of high-quality coordination in 2030. Beijing and Zhejiang will also reach good coordination, while Liaoning, Shanghai and Hainan will maintain growth at a slow rate of improvement. Notably, the coupling coordination degree in Tianjin will decrease from mild to moderate maladjustment, which is worthy of further consideration.

### Suggestions

#### Improve the coupling power of national fitness and national health

The coupling coordination degree of national fitness and national health in China is currently in its primary stage and, accordingly, needs guidance. The national fitness and national health strategy has risen to the level of a national strategy. However, the corresponding policies and regulations are still very weak, and the specific implementation rules have not yet been promulgated. China is advised to strengthen its top-level design at the policy level, improve the specific implementation rules and strengthen policy implementation. Government departments and managers of national fitness and national health activities must strengthen the mutual cooperation between sports and health departments and form a sports health promotion model of both vertical and horizontal collaborative innovation and cross-domain governance. At the same time, while making rational use of existing resources, the government and society must increase their capital investment, build sports venues and facilities and train talent at the intersection of sports and medicine. All these measures are bound to provide a continuous driving force for the coupling of national fitness and national health.

#### Strengthen the driving effect of national fitness on national health

National fitness has a relatively weak effect on the development of national health in Eastern China; in turn, national health provides strong support for the development of national fitness. Therefore, the driving role of national fitness in national health must be strengthened, and the significance and fundamental purpose of raising national fitness to the level of a national strategy need strengthening. Undeniably, a large proportion of Chinese people have an insufficient understanding of the concept and the internal mechanisms of national fitness, and the concept of emphasizing medical care and neglecting sports still occupies a dominant position among members of the public. The lack of willingness to take the initiative to prevent disease and promote health through nonmedical sports intervention indirectly implies that it is necessary to establish the concept of great health, to change treatment to focus on people’s health at the center and to give full play to the unique advantages of fitness in promoting a healthy and happy life for people. Extensive publicity on the great significance of national fitness with the theme of sports and health, the popularization of sports fitness knowledge, the promotion of scientific fitness methods, the creation of a social atmosphere for everyone to participate in physical exercise, and the consolidation of the mass foundation of national fitness are necessary. These measures can further enhance people’s enthusiasm to participate in national fitness, consciously strengthen their physical fitness and gradually achieve national health through the popularization and development of national fitness.

#### Strengthen cross-regional cooperation and achieve coordinated development

This study suggests that relevant departments should pay more attention to the spatial differences in the coupling and coordinated development of national fitness and national health in Eastern China. Cross-regional cooperation among provinces and cities must be strengthened to achieve resource sharing, complementary advantages and win–win cooperation. In October 2020, Shanghai, Jiangsu, Zhejiang and Anhui Provinces jointly issued some opinions on the integrated and high-quality development of sports in the Yangtze River Delta, which clearly pointed out the necessity to gradually promote the equalization of basic public sports services in the Yangtze River Delta region and to promote the deep integration of national fitness and national health. This document serves as a guideline for the comprehensive and coordinated development of sports in the Yangtze River Delta region to truly realize mutual reference, the sharing of resources, the coeducation of talent and the sharing of achievements. In addition, the direction for the cross-regional cooperation and development of national fitness and national health is defined. Therefore, other provinces may learn from this regional sports integration development system and path model to promote their coordinated development of regional national fitness and national health.

## Conclusion

The coupling and coordinated development of the two systems of national fitness and national health not only plays a vital role in the realization of national health but also highly promotes the development of national fitness work. First, this study constructs a coupling index system for national fitness and national health. Subsequently, the coupling coordination relationship between national fitness and national health in Eastern China is empirically analyzed by combining the entropy weight method and a coupling coordination model. Finally, the development of the coupling coordination degree of the two systems is predicted by using DPS analysis software on the basis of the gray GM(1.1) prediction model. The results suggest the following. First, national fitness and national health in Eastern China show an apparent interaction, with the characteristics of coupling and coordinated development. Second, the development level of national fitness and national health systems in various provinces and cities in Eastern China shows an overall upward trend and high correlation. Compared with the comprehensive level of national health development, the national fitness system is more volatile and is more likely to become an effective way to narrow the differences in development between regions. In addition, national fitness has a weak driving effect on national health, which in turn plays an important role in promoting the development of national fitness. Third, in terms of the relative development types of national fitness and national health, on the whole, the number of provinces in Eastern China that lag in national fitness is clearly more than the number of provinces that lag in national health. Tianjin, Hebei, Liaoning, Shanghai, Zhejiang, Shandong and Hainan all lag in national fitness, while Beijing, Jiangsu, Fujian and Guangdong show the coexistence of a lag in national fitness and a lag in national health. Fourth, the coupling coordination degree of national fitness and national health in various provinces and cities in Eastern China is increasing steadily year by year. In addition, the coupling coordination degree rises from barely coordinated to primary coordination, with a large spatial difference in the coupling coordination degree. From the perspective of the spatial correlation effect, no spatial aggregation occurs in the coupling coordination degree of national fitness and national health among provinces and cities in Eastern China. The spatial spillover effect is insufficient, as is the promoting effect among neighboring provinces. Fifth, according to the forecast results, the coupling coordination degrees of provinces and cities in Eastern China are expected to accelerate in the next 10 years, but spatial differences may remain.

## Limitations and prospects

In this study, the coupling coordination degree of national fitness and national health is determined on the basis of a multi-index evaluation system, which may have limitations in its construction. The construction of the index system cannot fully reflect the comprehensive level of national fitness and national health because the current national statistical indicators related to national fitness and national health are relatively singular. With the continuous optimization of and improvement in the statistical system related to national fitness and national health in China, a more scientific, systematic and useful index system can be constructed in the future to considerably enhance the credibility of the research results. Second, this study takes the eastern region of China as the sample for analysis. A more detailed division method, such as a study on the coupling and coordination of national fitness and national health in prefectures or cities, could provide more meaningful application value for local governments at all levels to promote the organic integration of national fitness and national health. In addition, data and energy constraints led this study to analyze panel data from only 2016 to 2020. Future studies are recommended to collect more data and conduct more in-depth research over a greater span of time.

## Supporting information

S1 Data (XLSX)
